# The Hygiene of the Skin, or the Art of Preventing Skin Diseases

**Published:** 1888-04

**Authors:** 


**The Hygiene of the Skin, or the Art of Preventing Skin Diseases.** By A. RAVOGLI, M. D., Cincinnati. Central Medical Publishing Co., 1888. Price, $3.00.

This is essentially a treatise on diseases of the skin, in which particular attention is given to etiology and prevention. It contains much valuable matter not found in the numerous excellent works already in the hands of the profession. It is a book of over 400 pages, and in this necessarily short notice we can perhaps give our readers the best knowledge of its value by giving the titles of its chapters. Chapters I. to IV. are devoted to the skin, its anatomy, its functions, and the effect of morbid impressions. Chapter V. is devoted to the diatheses, and

VI. to the skin diseases which are produced by a particular virus or virulent impression. VII., VIII. and IX. consider physiological individual conditions, Eruptions produced by remedial or poisonous substances. Influence of diet, External causes of skin disease, Influence of water upon the health of the skin, Cosmetics, Influence of clothing in the production of skin diseases, the Hair, and a chapter on the Parasites, complete the titles of the eighteen chapters of the book. The illustrations are fairly good, and the work, as a whole, is deserving of commendation.

